# Evaluating Health Care Provider Perspectives on the Use of Mobile Apps to Support Patients With Heart Failure Management: Qualitative Descriptive Study

**DOI:** 10.2196/40546

**Published:** 2022-10-26

**Authors:** Bridve Sivakumar, Manon Lemonde, Matthew Stein, Sarah Goldstein, Susanna Mak, JoAnne Arcand

**Affiliations:** 1 Faculty of Health Science Ontario Tech University Oshawa, ON Canada; 2 Social Research Centre Ontario Tech University Oshawa, ON Canada; 3 School of Nutrition, Faculty of Community Services Toronto Metropolitan University Toronto, ON Canada; 4 Division of Cardiology Department of Medicine University of Toronto Toronto, ON Canada; 5 Department of Medicine Sinai Health Toronto, ON Canada

**Keywords:** heart failure, mobile health, mHealth, eHealth, mobile apps, adherence, self-management, mobile phone

## Abstract

**Background:**

Nonadherence to diet and medical therapies in heart failure (HF) contributes to poor HF outcomes. Mobile apps may be a promising way to improve adherence because they increase knowledge and behavior change via education and monitoring. Well-designed apps with input from health care providers (HCPs) can lead to successful adoption of such apps in practice. However, little is known about HCPs’ perspectives on the use of mobile apps to support HF management.

**Objective:**

The aim of this study is to determine HCPs’ perspectives (needs, motivations, and challenges) on the use of mobile apps to support patients with HF management.

**Methods:**

A qualitative descriptive study using one-on-one semistructured interviews, informed by the diffusion of innovation theory, was conducted among HF HCPs, including cardiologists, nurses, and nurse practitioners. Transcripts were independently coded by 2 researchers and analyzed using content analysis.

**Results:**

The 21 HCPs (cardiologists: n=8, 38%; nurses: n=6, 29%; and nurse practitioners: n=7, 33%) identified challenges and opportunities for app adoption across 5 themes: *participant-perceived factors that affect app adoption*—these include patient age, technology savviness, technology access, and ease of use; *improved delivery of care*—apps can support remote care; collect, share, and assess health information; identify adverse events; prevent hospitalizations; and limit clinic visits; *facilitating patient engagement in care*—apps can provide feedback and reinforcement, facilitate connection and communication between patients and their HCPs, support monitoring, and track self-care; *providing patient support through education*—apps can provide HF-related information (ie, diet and medications); and *participant views on app features for their patients*—HCPs felt that useful apps would have reminders and alarms and participative elements (gamification, food scanner, and quizzes).

**Conclusions:**

HCPs had positive views on the use of mobile apps to support patients with HF management. These findings can inform effective development and implementation strategies of HF management apps in clinical practice.

## Introduction

### Background

Heart failure (HF) is a progressive clinical syndrome in which abnormalities in heart function, marked by reduced cardiac output and congestion [1], often result in periods of acute decompensation. HF is managed through pharmacological therapies, accompanied by self-care recommendations that emphasize dietary modification and daily weight and symptom monitoring [1,2]. However, patient adherence to these treatments can be challenging, with medication and dietary nonadherence rates being 50% and 22% to 50%, respectively [3-7]. Nonadherence is associated with increased risk of HF hospitalizations and mortality, which contribute to the growing economic burden of HF [8].

Currently, a significant amount of behavioral and nutritional counseling occurs in the clinical setting, with the counseling provided by health care providers (HCPs), including physicians and nurses, with consultation from pharmacists and registered dietitians as needed, to support patients with HF management and adherence [9-12]. The delivery of this education can be limited by HCPs’ lack of knowledge, time, and compensation [13]. In addition, patients with HF, especially those living in rural and remote regions, may not have access to these professionals, and even if they do, HCPs are unable to monitor the patients and provide feedback in real time and on day-to-day progress. Given the clinical relevance of treatment adherence to HF outcomes and the real-life challenges that patients may experience, it is not surprising that initiatives to support adherence are highlighted as a priority action area by the American Heart Association [14].

Mobile health (mHealth) technologies present opportunities to improve adherence and support HF management. Several randomized controlled trials examining the impact of mHealth-based interventions in HF have reported significant improvements in cardiovascular and all-cause mortality, New York Heart Association class, left ventricular ejection fraction**,** quality of life, and physical functioning [15-18]. mHealth-based interventions improve outcomes by supporting the delivery and continuity of care in HF by relaying health information, monitoring patient symptoms outside of clinical setting, and supporting patient education [19,20]. Modern mHealth tools such as mobile apps are also able to provide real-time feedback in a way that is less resource intensive than other eHealth interventions (eg, telemonitoring) [19,20]. Multiple systematic reviews have reported that mHealth apps for HF improve engagement in self-care behaviors as well as patient self-efficacy, self-confidence, and communication with HCPs, offering a potential cost-effective solution to support patients with HF treatment adherence and self-management [21-24].

### Objectives

Among existing apps available to support HF management, few are considered high quality based on content, features, and functionality when assessed against established rating scales [24,25]. In fact, it has been suggested that many apps require redesign because of a lack of appropriate features to engage patients in self-care and failure to meet the needs and motivations of the population with HF [24]. In contrast, well-designed mHealth apps that integrate input from both patients and HCPs are more likely to also meet HCPs’ needs, leading to overall better acceptability and HCPs’ willingness to adopt and recommend such tools to their patients [26]. Moreover, HCPs have a unique understanding of what is required to support patients in HF management [27]. A few studies have explored HCPs’ perceptions on the use of technology-based interventions for HF management [28-30]. However, these studies have only focused on mobile phone–based interventions for wireless Bluetooth-enabled remote monitoring of patient symptoms, SMS text messaging, and sensor-focused mHealth apps and do not capture HCPs’ perceptions on mHealth interventions using more advanced applications, which have unique opportunities and challenges of their own. Determining HCPs’ perspectives and attitudes on the use of mobile apps for HF management can inform the effective design of such apps, including their features and content, increasing the likelihood of app adoption in this population. Therefore, the objective of this qualitative descriptive study was to determine HCPs’ perspectives (needs, motivations, and challenges) on the use of mHealth apps to support patients with HF management. For the purposes of this study, HCPs included cardiologists, nurses, and nurse practitioners.

## Methods

### Study Design and Research Team

This study followed a qualitative descriptive design. Rooted in naturalist inquiry, this design allows for meaningful summarization of the data in everyday terms and has been used to inform development of health interventions [31]. The study followed the COREQ (Consolidated Criteria for Reporting Qualitative Research) guidelines for qualitative research [32]. The research team included a PhD graduate student (BS); 2 faculty members with expertise in HF, digital intervention research, and qualitative methods (JA and ML); an HF cardiologist (SM); and a social scientist with qualitative expertise (MS). There was no prior relationship between the interviewer and the participants.

### Ethics Approval

The study was approved by the research ethics board of Ontario Tech University (14882), and informed consent was obtained from all participants.

### Study Participants and Recruitment

Purposive sampling was used to recruit cardiologists, nurses, and nurse practitioners who work in outpatient HF programs in Canada. Registered dietitians and pharmacists were excluded because they are not the primary point of care for patients with HF. Recruitment was conducted with advertisements and emails circulated by the Canadian Heart Failure Society as well as with a snowball sampling approach. Eligible participants were invited to participate via an email invitation. Participants completed a web-based consent form. Participants were compensated CAD $20 (US $15) in the form of a gift card for their participation in the study.

### Data Collection

One-on-one 15-minute telephone interviews were conducted in English with participants between February 4, 2019, and June 4, 2020. Telephone interviews allow for flexibility and convenience for both researchers and participants and is an acceptable method for qualitative data collection [33,34]. The interviewer (BS) recorded field notes during and after each interview, which included reflective memos on unique ideas and insights as well as their interview experience. Participants were sent the interview questions before the interview.

The interviews were directed by a semistructured interview guide (Multimedia Appendix 1) that consisted of 8 open-ended questions that reflected the aim of the study. These questions were supplemented with research probes and paraphrasing to generate further clarification of participant responses and promote discussion. The interview guide was developed by expert consensus and informed by the diffusion of innovation theory [35], which is widely used in guiding the development and evaluation of innovations. The interview questions reflected the five main factors that influence the adoption of an innovation: (1) relative advantage refers to the degree to which an innovation is seen as better than standard care, (2) compatibility refers to how consistent the innovation is with the needs and values of the adopter (eg, patients), (3) complexity refers to the difficulty of the innovation, (4) trialability refers to the extent to which the innovation can be tested before use by users, and (5) observability refers to the extent to which the innovation provides results. The interview guide was reviewed and approved by members of the research team to ensure clarity and appropriateness of questions and probes. The guide was pilot-tested with an HF cardiologist external to the research team.

The interviews were audio recorded using a voice recorder, and the recordings were manually transcribed verbatim (BS and SG). Pseudonyms were used in the transcripts to protect the identity and maintain anonymity of the participants. All identifiable information was removed from the transcripts. The verbatim transcripts were verified by a research assistant by comparing the transcripts with the audio recordings to ensure accuracy.

Before the telephone interview, participants completed a short web-based questionnaire that asked about their views on using technology for managing HF as well as barriers related to supporting patients’ diet and medication adherence. The questions were informed by what is known in the literature about barriers and facilitators related to medication and dietary adherence. The questionnaire consisted of 10 Likert scale–style questions, with answer choices ranging from 0 to 3 (I don’t know, agree, neutral, and disagree). Sociodemographic information, including age, sex, years of practice, and professional role, was also collected. The questionnaire was validated by the research team for face and content validity.

### Data Analysis

Preceding analyses, all participants received their transcript for *member checking*, as described by Lincoln and Guba [36], to approve the verbatim transcripts and verify accuracy. Only minor amendments were received and integrated into the final transcripts, ensuring credibility of data. To prepare for data analysis, the audio recording, transcript, and field notes of each interview were reviewed multiple times. The transcripts were imported into NVivo software (version 12.0; QSR International), which supported the content analysis. The transcripts were inductively coded by 2 independent researchers (BS and MS). This was followed by comparison of coding, collaborative discussion of codes (for intercoder agreement), expansion of codes to capture subcodes, and ultimately the grouping of codes into common themes. For the purpose of this study, a theme reflected participant accounts related to their views (needs, motivations, and challenges) regarding the use of mobile apps for HF management. Themes were reviewed and finalized in discussion with a qualitative expert on the research team (ML) as well as the principal investigator (JA).

The questionnaire data were summarized using descriptive statistics. Categorical variables were presented as frequencies and percentages, and continuous variables were described as means and SDs.

## Results

### Overview

A total of 21 HCPs (cardiologists: n=8, 38%; nurses: n=6, 29%; and nurse practitioners: n=7, 33%) participated. The mean age of the participants was 42.9 (SD 8.6) years, and 81% (17/21) were women. Participants’ years of practice as HCPs included 1 to 5 years (1/21, 5%), 6 to 10 years (6/21, 29%), 11 to 15 years (5/21, 24%), 16 to 20 years (1/21, 5%), and >20 years (8/21, 38%).

**Figure 1:**
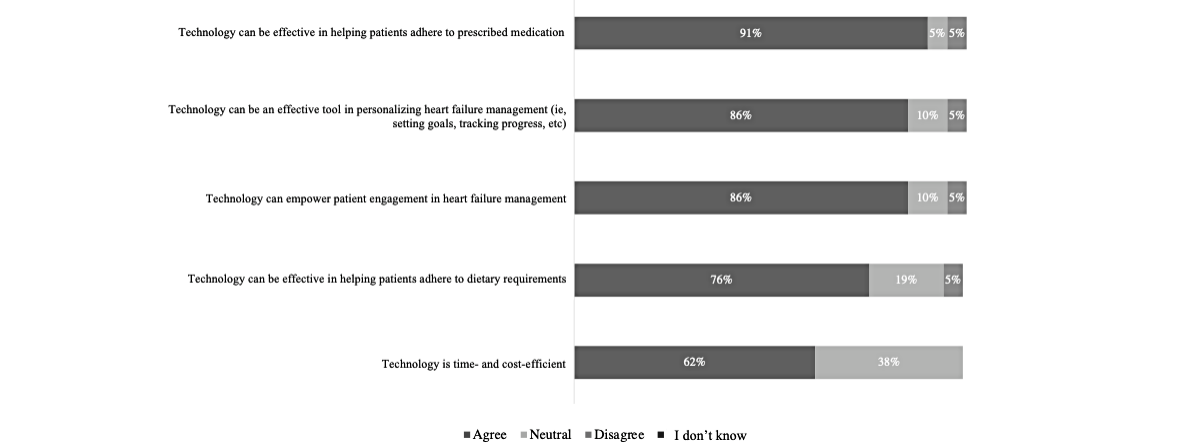
Health care providers’ views on the use of technology to aid in managing heart failure (N=21).

**Figure 2:**
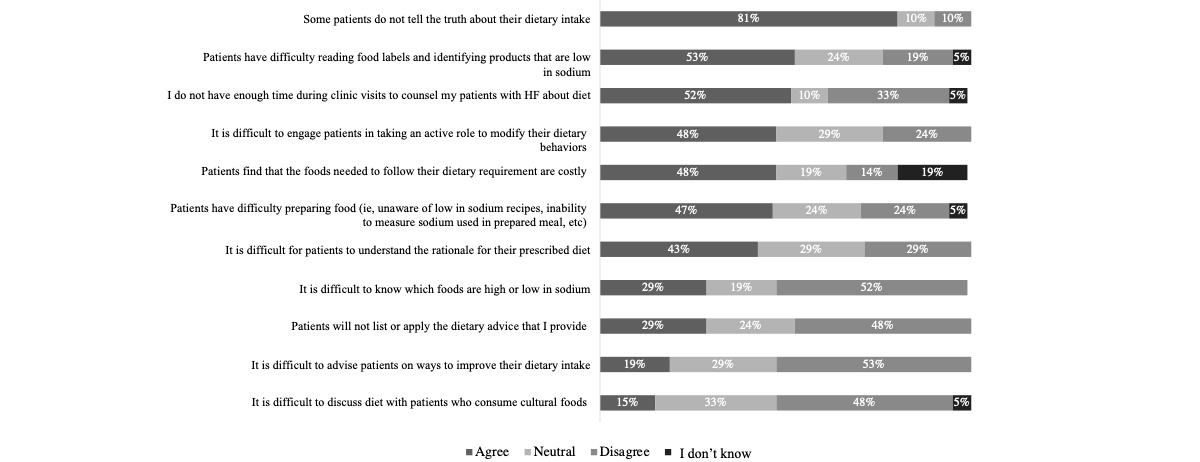
Barriers faced by health care providers when supporting patients’ dietary adherence (N=21).

**Figure 3:**
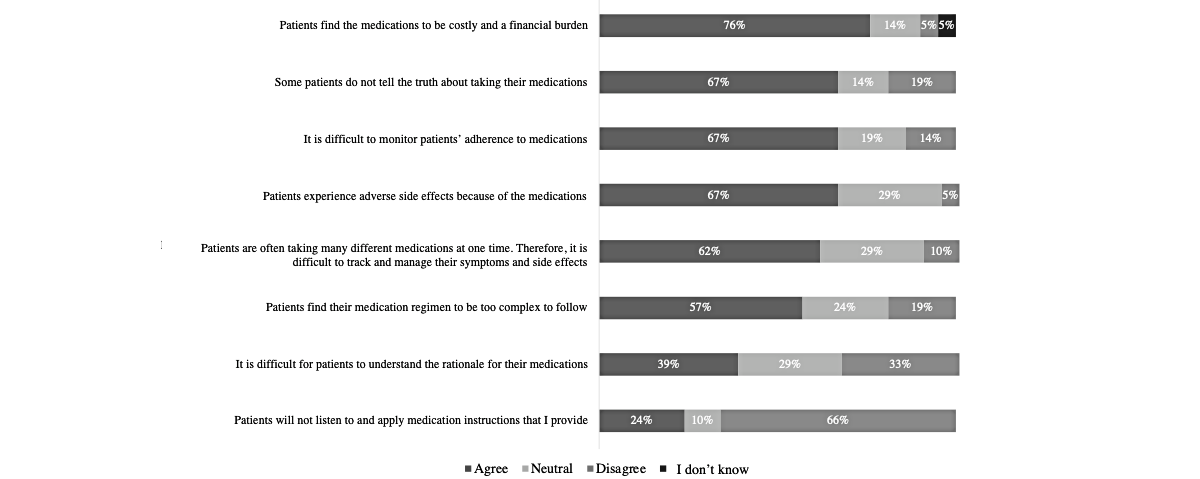
Barriers faced by health care providers when supporting patients’ medication adherence (N=21).

The questionnaire data indicated that the HCPs agreed that technology can be effective in helping patients to adhere to their prescribed medications (19/21, 90%) and dietary requirements (16/21, 76%; Figure 1). Barriers to supporting patients’ diet and medication adherence included medication cost and financial burden (16/21, 76%), difficulties with reading food labels and identifying low-sodium products (11/21, 52%), and patients not being truthful about taking their medications (14/21, 67%) or their dietary intake (17/21, 81%; Figures 2 and 3).

Five themes were identified from the telephone interviews that reflect participant perspectives on the use of mobile apps for HF management. These included participant-perceived factors that affect app adoption, improved delivery of care, facilitating patient engagement in care, providing patient support through education, and participant views on app features for their patients.

### Participant-Perceived Factors That Affect App Adoption

#### Patient-Related Factors Affecting App Adoption

Participants described several factors that may affect the use of mobile apps by patients with HF, including *patient age, access to mobile phones and internet, how* technology savvy *patients are, physical and cognitive function of patients,* and *their level of engagement in HF self-management.* Participants viewed apps as being more favorable among “younger” patients with HF, suggesting that the majority of patients with HF were older adults (aged >70 years), and thus they would be unfamiliar with using technology. One participant stated as follows:

I don’t like to peg people into categories, but certainly it seems like the younger crowd for which is like 50s, 60s, they might be the ones more interested in using [apps]. We certainly have a high number of elderly, or frail elderly in our clinic so a lot of them aren’t, you know, on email or internet or things like that.

Participant 9

In addition, characteristics associated with aging, such as decline in physical and cognitive functioning, were considered barriers to app use. There was also concern that patients may lack access to mobile phones and internet needed to use apps, particularly in northern and rural communities. Participants expressed that some patients may lack motor function, have arthritis, be missing digits, have difficulty with their vision, or experience cognitive challenges, which would impede their ability to use apps. Participants also identified that adoption of apps required a certain level of motivation from patients and that those who are highly engaged in HF self-management would likely benefit from using such tools.

#### HCP-Related Factors Affecting App Adoption

HCPs’ *buy-in* and *familiarity with HF apps* as well as *level of time, compensation,* and *workload burden for HCPs* were perceived as factors that may affect their app use. Although some of the participants had experience using mobile apps in their clinical practice to monitor patient care, the majority were unaware of credible HF management apps to recommend to their patients. Moreover, buy-in from HCPs and clinical staff may be a challenge to app adoption in the clinical setting. A participant observed as follows:

I don’t know how we’ve gotten to this place but so many people are, they are negative nellies. They are not willing to try new things, because “oh, it’s not going to work,” “oh I’ve seen this, it’s not going to work.” How do we know unless we try? It’s something new. Technology is where it’s at, we all know that. So, I think the buy-in from staff is going to be part of the challenge.

Participant 11

Participants indicated that the time and workload required to teach patients how to use the app, interacting with patients through an app interface, and interpreting patient data from an app may interfere with app use. If app use was time consuming, it was felt that a lack of compensation for their time can prevent HCPs from using apps in their clinical practice. One participant stated as follows:

If I need to spend hours in each clinic appointment educating the patient on how to use it [app], it’s going to fall at the first hurdle. I don’t have the time; I don’t have the money.

Participant 13

#### App-Related Factors Affecting App Adoption

The perceived app-related factors affecting adoption included *information provided by apps, user-friendliness of apps, level of technical support and guidance provided for app use, app availability across multiple devices, level of privacy and protection for patient information,* and *integration into clinical practice and health care system* as well as *language and costs associated with app use.* Participants felt that apps providing personalized and tailored information to patients were valuable compared with apps presenting generalized information about HF. Apps also need to provide simple, practical, and meaningful information as well as be easy to use, simple, user-friendly, and compatible with different types of devices and platforms (smartphones, tablet devices, and web). Moreover, technical support and reasonable support and guidance on how to navigate the app should be provided. Participants felt that HF apps need to be encrypted, safe, and secure to ensure confidentiality of personal information. In addition, it would be beneficial to have apps be integrated into practice and the health care system, including the hospital, care team, and electronic medical records. A participant made the following observation:

Now to use it [app], our whole team would have to adopt it. Meaning they would have to have a consensus on its use and then if we wanted to have data sent to us then obviously, that would be a whole system, how do you receive this information, how do you use this information, what’s the protocol for receiving it and then acting on it, type of thing.

Participant 6

By contrast, some factors were identified as barriers to app use. Participants felt that language can be a barrier because not all patients with HF may be comfortable using apps that are primarily in English. Costs involved in downloading and using apps were also seen as a barrier. In addition, participants were concerned that the use of technologies such as apps may promote the use of appointments via telephone or videoconference, which they feel can be challenging because of the lack of in-person interaction.

### Improved Delivery of Care

Most participants felt that apps may positively affect their *ability to provide remote and timely care*, including remote monitoring, titration of medications, and check-ups, all of which may allow for timely delivery of care. It was also viewed that by providing opportunities for remote care, apps could *limit clinic visits and save patient time and resources,* including travel and parking costs associated with clinic visits. It was expressed that sometimes patients face difficulties with scheduling and clinic appointment travel; thus, apps may make care more accessible. One HCP provided an example of how an app supported remote care in their practice:

Well for example I have a specific patient that is on [name of app]...cardiac failure that is related to myeloma, but [patient] is very sick and is on chemotherapy so by using the app I have been able to keep [patient] at home without coming to hospital. I don’t know how much time they have, but the family is really happy that [patient] stays at home. And we have been asking for weight changes very quickly to try to keep them at home.

Participant 1

Apps may also provide additional benefits when compared with traditional telehealth services. A participant expressed the following view:

The benefit is for sure we have maybe more information than the usual phone call. So, if you can incorporate things that the patient can, let’s say, send a picture or sharing how they look like. So, we have that visual, you know, presentation in front of you.

Participant 7

In addition, they can potentially *collect and assess real-time health information and prevent adverse events.* Participants felt that apps can collect real-time and day-to-day data on HF signs and symptoms that can be shared with HCPs, as needed. Specific data considered important by participants were weight, blood pressure, common HF symptoms (ie, swelling and shortness of breath), step count, and daily sodium and fluid intake. Apps could allow HCPs to gauge trends in these data to better assess the patient. An HCP stated as follows:

It’s also helpful to have the data when the patient comes to clinic because we can clearly sort of go back and say, “hey you know, this is what trend of this vital sign has been” and that’s helpful information when you’re seeing someone.

Participant 3

Participants felt that, ultimately, by being able to collect, share, and assess health information remotely, apps have the potential to identify worsening clinical signs and symptoms and precipitating factors for adverse clinical events, allowing for early intervention and the prevention of HF hospitalizations. A participant made the following observation:

The device would let the attending physicians know when the patient was not doing that well...maybe some complications could be caught on time before they got really sick.

Participant 17

In addition, it was perceived that apps that use artificial intelligence could alert HCPs of patients who require immediate care. One HCP stated as follows:

...patient-reported symptoms that are algorithmically determined at which point they create an alert...so you know worsening clinical symptoms create an alert that alert is then sent to a nurse or physician.

Participant 15

### Facilitating Patient Engagement in Care

Participants felt that apps can be used to *foster independence, awareness, and confidence* among patients because they can *support establishment of health goals and provide feedback and reinforcement.* Apps could encourage patients to take “ownership of their disease” and “empower” them to engage in self-care activities. In addition, they can allow patients to have awareness of their disease and health status. It was expressed that apps can support patients in goal setting and “guide them to make SMART goals” (ie, goals that are specific, measurable, achievable, relevant, and timely), as well as challenge patients in improving their health behaviors over time. A participant noted as follows:

I think it’s a great example that maybe you set a goal, okay so yesterday I can walk about a block before I’m getting shortness of breath maybe today let me try to walk one and a quarter block and see how I feel something like that.

Participant 18

Moreover, participants felt that apps present an opportunity for patients to “have ongoing reinforcement of the heart failure education of the diet and medication that are recommended for them.” Apps can also generate automated feedback for patients based on their HF symptoms and specific medication and dietary intake behaviors that would otherwise be difficult to provide during clinic visits. Participants made the following observations:

...so that way they would have feedback, you know, “this week you actually missed your medication three times.” Perhaps prompting with a screen that says “you require a compliance of at least 90% to see effectiveness in this goal, this goal and this goal.” So, kind of providing them with some research feedback.

Participant 21

...you can say did you know that the choices, the ones you made are more higher in salt or you know you can give them more feedback, that structure is hard to do one on one in person visit.

Participant 19

Several participants thought that app adoption for HF management can *facilitate connection and communication between patients and their HCPs*. This could include the incorporation of a messaging feature for patients and HCPs, which can serve as a more efficient communication method than traditional telephone calls. An HCP commented as follows:

...having the possibility of communicating with patients outside of phone call could be very helpful, a way to just send message that could be faster than having us to call back to answer questions or to confirm an information.

Participant 4

This type of communication may allow patients to write down questions in real time and engage in back-to-back communication with their HCPs, which can lead to more open conversations about their care. A participant observed as follows:

Also, it may allow patients to kind of write down questions or they may be more open to discussing, what their intents are in the written form as opposed to face-to-face and they don’t have to think about it. So, it’s that extra time, it’s not done by, for instance, where they have messages that go back and forth or what have you. It can allow them to kind of open up more, to think more about what they want to ask, and what kind of care do they want to have in the future.

Participant 5

One of the frequently mentioned opportunities for the use of HF mobile apps was that they can help patients *monitor and track self-care activities and indicators.* Participants felt that it may be beneficial if HF apps allowed patients to track their diet, including sodium and fluid intake, through manually entering food intake and scanning food labels. Participants stated as follows:

I think what would be very valuable is a way to actually track, the way that um the way that weight loss apps track you sort of have an ongoing diary of how much you eat and then it spits out your calories similarly, I think having um an ongoing tracking system of fluid intake would be particularly useful.

Participant 15

So, I think putting in milligrams of sodium is a nice way in some kind of visual way where a lot of what looks like a battery and it’s full at the beginning of the day and that represents your 2000 mg and then you have breakfast and you can sort of calculate it, depending on how good or bad you are at that, and then it’s going to deplete some of that energy or sodium allowance per se.

Participant 6

In addition to tracking dietary intake, participants also saw opportunity for an app to track patients’ physical activity (ie, step count) as well as patient symptoms (ie, daily weight, blood pressure, and pulse). It was suggested that an app for HF can be linked directly to other apps that track symptoms, diet, or exercise (eg, MyFitnessPal), allowing patients to have all information in “one spot.”

### Providing Patient Support Through Education

Apps were viewed as a medium for patients to obtain *access to resources*. This included information about HF, HF guidelines and symptoms, and mental health support. Some of the participants felt that HF management resources available on the web (eg, the Heart and Stroke Foundation of Canada website) would be beneficial to patients in an app form because apps can present information in a more engaging way through visuals, interactions, and videos. One participant saw a unique opportunity for apps to tailor patient resources based on their geographical location:

I was thinking about an app and how great it would be if that app you could plug in your geographic location and it would give you local access or national online access to information on any types of events, webinars, support groups.

Participant 6

A commonly perceived benefit to using an app for HF management was the potential for apps to facilitate *nutrition-related education.* As sodium restriction was a focal point of dietary education for HF management, it was felt that an app could teach patients about sodium intake recommendations, common dietary sources of sodium, and the sodium content in foods. Dietary potassium was also identified as an important part of education for patients with HF. Participants made the following observations:

Salt restriction, giving them an idea of you know how much salt is recommended and then an example of what you know I always give this example to my patients the limitation is 2000 mg a day for the cardiac and a dill pickle is 550 so a quarter of the salt intake is in one dill pickle. So, it gives them real perspective.

Participant 16

...kind of a dictionary where they could enter the name of food and see how much sodium...so they could see that oh well a bag of pretzel is 2 grams of salt and realize oh no I should not eat that because of the salt in it.

Participant 4

...foods that would be high in potassium, so sometimes our patients have higher potassium that limits our ability to get them on guideline directed medical therapy or up-titrated and so knowing what foods were higher in potassium might be helpful because if we said to the patient “we want you to eat foods that are lower in potassium,” they always want to know what those are.

Participant 3

Apps could also support food skills development such as reading food labels on packaged foods, food preparation, and “culinary literacy.” It was also identified by several participants that apps can have information to guide patients about foods to consume versus foods to avoid, as well as provide dietary tips (eg, managing sodium intake on cheat days and managing dry mouth), acceptable low-sodium substitutions, and low-sodium recipes:

But if there would be little tips and tricks on things, if you have to buy canned green beans just rinse them off. Get rid of a lot of that sodium. Have tips like that on there...I think would be very helpful for people.

Participant 11

*Providing access to medication-related information* was another perceived benefit of apps. Multiple participants identified that patients may be better adherent to their HF medications if they are aware of the purpose for which the medications are prescribed and the “risk of skipping a couple of doses”:

...you know, in a much more basic level, why you are taking these drugs and why you are not to stop your ramipril just because your systolic only 100, you feel fine and you’re not dizzy and you keep taking it because it’s not for blood pressure...so having a bit of content in the background of why these medications are helpful, I think would be a little bit important for content inclusion for an app.

Participant 6

...benefits of the medication, like a little blurb on why it is important that you take this medication, and all the ACE [angiotensin-converting enzyme] inhibitors decrease the mortality of HF by 30%, those things help the patient to be compliant.

Participant 4

Other medication-related information considered beneficial included a personalized medication list with relevant information such as name (ie, brand and generic drug names) and dosage as well as information on medication interactions and side effects for prescribed and over-the-counter medications. Participants stated as follows:

Common side effects that they may anticipate, from the different families. You know they can go into ACE [angiotensin-converting enzyme] inhibitors, beta blockers, MRAs [mineralocorticoid receptor antagonists], they can go with all those different categories and look at, and self-education about the medication they are on so they know about it and know what side effects could possibly come up.

Participant 8

...interaction of medications is always a useful thing to have, especially over the counter and the normal medication.

Participant 5

### Participant Views on App Features for Their Patients

App features perceived by participants as useful for patients included g*amification*, *reminders and alarms*, and *food scanners*. Participants had mixed views on the gamification of apps. Some of them felt that gamifying an app for HF may appeal to certain patients and if designed well it can be “fun,” “enjoyable,” “engaging,” and “interactive,” which can promote learning. One such example was embedding quizzes into the app that test patient knowledge on disease management. Other participants questioned the benefits to incentivizing someone’s health and whether patients would want to play a game, viewing it as an added task. A participant stated that they had “never seen a successful cardiovascular gamification in an app.” It was noted that gamification can be an attractive feature, especially for “young” patients with HF; however, it may be “demeaning” or not suitable for patients with HF, who are on average older. The HCPs felt that if an app for HF were gamified, it needs to be designed in a “mature” way. Participants made the following observations:

Obviously like, interaction helps to promote education at all ages. You might have a hard time getting buy in from the older population. Just because you get that “I’ve been alive for 80 years I know what I am doing” type of thing. But that’s okay it’s never perfect for everybody. But I think would help with engagement.

Participant 6

I think it’s great. My kids use educational apps that are kind of in a game format. So, I think it has a purpose. The question is, how do you do it for a mature adult? You know, are they going to find it too childish or are they going to actually enjoy it? If it’s done well, I think it’s great to keep patients engaged potentially.

Participant 5

You know our population is elderly, they are frail, English is another language of theirs, they are hard of hearing, they are visually challenged. So, yeah. I mean they are not playing cards on their phones.

Participant 20

Participants expressed that integrating reminders and alarms in an app to reinforce daily weighing, fluid intake, and exercise as well as prescription refills and physician’s appointments would be helpful. Nearly all participants agreed that reminders in an app for taking pills would be useful for patients, with some suggesting the option for patients to personalize the reminders and alarms (ie, turn them off). It was mentioned that patients are often prescribed multiple medications, to be taken multiple times a day, for their HF as well as other comorbidities; thus, they may have difficulties with medication adherence because of forgetfulness. One HCP commented as follows:

I was thinking about the medications. Like trying to make it compliant for the patients with their medications. If there was some sort of alarm, you know, within the app that would automatically remind them: “Okay it’s time to take your pills.”

Participant 11

Another feature that participants considered useful in an app was the integration of a food scanner, whereby patients can take a picture of their food plate or scan a food label (ie, nutritional facts table), which will then display nutritional information such as sodium content and calories:

I think it’s important, you know, the scanning of labels and then that calculates your salt content based on serving size and that would be a visual reminder of how much salt is actually in that and I think that when people scan enough labels, they’ll realize what they can and cannot eat.

Participant 14

## Discussion

### Principal Findings

To our knowledge, this is one of the first studies to determine the perspectives of HF cardiologists, nurses, and nurse practitioners on the use of mobile apps for HF management. Overall, participants had positive views about using mobile apps to support their patients with managing HF. They identified factors affecting app adoption (eg, patient age and technology access) and opportunities for app use, such as improving delivery of care, providing patient support through education, and facilitating patient engagement in care. App features such as gamification and quizzes were also identified by participants as being useful for patients. Our findings support previous research reporting that mHealth apps have the potential to be cost-effective interventions that optimize provision of care and support patients in HF self-management [22,25].

Perceived factors affecting app adoption related to the patient, clinical practice setting, and the apps themselves are consistent with findings from past studies [37-39]. One of the most frequently mentioned factors that affect app adoption, as perceived by our participants, was patient age. Findings from several studies also cite age as an individual-level factor affecting acceptance and use of mHealth apps [37,40-42] because most individuals using such technologies are often younger (aged <35 years), with those aged >70 years using mobile apps at the lowest frequency [40]. These findings are explained by Cajita et al [43] who found that older adults (aged ≥65 years) tended to lack knowledge on how to use mobile technologies. Evidence also suggests that older adults’ self-efficacy is low when learning to use mHealth apps [44]. Despite such findings, smartphone ownership among people with HF is relatively high among all age groups (eg, 84% in those aged 50-64 years), with older patients with HF also showing willingness to use mHealth apps to support HF management [45]. As the use of mHealth technologies for health-related activities is an emerging field, it is expected that older adults may face some difficulty and require support when using apps for HF management. However, the capability of patients with HF to use mobile apps should not be based on age alone; rather, factors associated with aging, such as visual impairment and cognitive dysfunction, may be more influential in the use of apps to support HF management. Regardless, accessibility features to accommodate users with special needs should be considered when designing and developing apps. It is imperative to keep this in mind to close the digital divide among older adults and promote more equitable use and distribution of mHealth technologies [43,45].

A lack of time and workload burden for HCPs as well as the ability of an app to be integrated into clinical workflows were identified as factors influencing app adoption for HF management, particularly for apps that have features for clinical monitoring and patient-clinician interaction. These data are supported by 2 recent reviews, where increased work and responsibilities as well as lack of integration with electronic medical records were among the most frequently identified clinician-level barriers to digital health adoption [38,39]. The majority of mHealth tools for HF management focus on telemonitoring, which HCPs consider to be time and resource intensive because these tools can produce more web-based data, additional administrative work, and increased communication and interactions with patients [29]. It is unknown whether mobile apps supporting various other functions such as education and behavior change have similar challenges within the context of HF management, although several of these workload concerns may be common when using technology for health care delivery in general. HCPs may also need to provide patients with training to support the use of mHealth tools, a concern identified by participants in our study. Evidence suggests that clinicians are less likely to adopt mHealth technologies if they believe that such tools do not reduce their workload [46,47]. This has direct impact on mHealth uptake among patients because patient adoption of these tools is often dependent on HCPs’ recommendations [48]. To address HCPs’ concerns regarding mHealth workload, it is imperative that they are recognized as stakeholders in mHealth technology development and implementation. In line with recommendations by Davis et al [49] and Radhakrishnan et al [50] for remote monitoring and telehealth technologies, we emphasize the importance of involving HCPs during the design, development, and implementation stages of mHealth apps to maximize the relevance and usability of such apps, which can result in overall better uptake and adoption. Moreover, practicing and in-training HCPs should receive adequate education on the use of digital health technologies [38] to increase their familiarity and comfort with such tools, which can increase their acceptance and uptake [51]. Proper integration with electronic medical records and clinical workflow can also facilitate mHealth app adoption [38,49].

Participants in this study saw several opportunities for using apps for HF management. Notably, participants felt that apps can support patients with HF by providing access to dietary and nutritional information as well as medication-related information. However, by contrast, most HF management apps are focused on daily monitoring of symptoms, with only a few addressing diet and medication [24,25,52]. Moreover, of the apps that include diet and medication, the focus is on tracking behaviors, and these apps fail to incorporate key diet- and treatment-related knowledge and skills, such as low-sodium diet and interpreting food labels as well as information on medication interactions and side effects, which are important features identified by participants in this study; for example, according to our questionnaire data, 52% (11/21) of the participants agreed that patients have difficulty reading food labels and identifying low-sodium products. Although these are not patient-reported data, this is an indication that HCPs see opportunities for mHealth apps beyond symptom monitoring. Albeit, such objective measures related to diet and medication would be supportive in promoting adherence, facilitating targeted behavior change, and supporting patients in forming fundamental skills and habits for managing HF.

This study uniquely explored HCP perceptions on features that may be useful to incorporate in an HF mobile app. One such feature that was widely discussed was gamification. Gamification is the use of game design mechanics in real-life, nongame environments [53]. The use of game techniques is an effective way to engage, motivate, and sustain health behavior change in individuals [54-56], and such techniques (eg, goal setting, reinforcement, and social connectivity) are closely related to proven health behavior change techniques [53]. However, the use of gamification in mHealth is an emerging concept and is being explored in the context of nutrition, physical activity, diabetes, mental health, and cardiovascular disease, including HF. The perspectives related to gamification for patients with HF in our study were mixed, with some of the participants recognizing that it can be an engaging and participative app feature and others questioning its appropriateness for the older population with HF. Interestingly, Radhakrishnan et al [57] conducted prototype testing of an HF mobile app integrated with contemporary game technology among older adults with HF (aged ≥55 years) and reported that the HF digital game was easy to play, enjoyable, and helpful in learning about HF and resulted in significant improvements in HF self-management knowledge. This study [57] and others [58-60] suggest the potential of gamification to be an effective medium to increase disease-related knowledge and support self-management of HF, even among older adults [61,62].

### Limitations

Our study includes potential limitations that warrant discussion. Although telephone interviews produce data comparable with those produced in face-to-face interviews, a few limitations to this method of qualitative interviewing exist, including the inability to observe and respond to visual cues, lack of contextual data, and potential challenges to establishing participant-interviewer rapport [33]. Despite these limitations, the use of telephone interviews was favorable in our study because it allowed for geographical flexibility and an efficient cost- and time-saving method that accommodated participants’ schedules. Moreover, a part of our data collection period coincided with the COVID-19 pandemic, which may have shifted HCPs’ perspectives on the use of technology in clinical practice because of the necessary transition to remote care and telehealth use. In addition, many of our participants were women, albeit the perspectives of cardiologists, nurses, and nurse practitioners were equally represented. The perspectives of HCPs in this study are limited to those of cardiologists, nurses, and nurse practitioners. We acknowledge that other health care professionals such as family physicians, dietitians, and pharmacists may hold different views. Finally, we recognize that our own beliefs and assumptions could have biased study findings; however, steps were taken to minimize these biases. These steps included expert review of the interview guide, use of multiple data sources (interview and questionnaire), field notes by the interviewer, and independent coding by 2 researchers. We have also presented participant quotes that substantiate our findings and interpretations.

### Conclusions

This study demonstrated that cardiologists, nurses, and nurse practitioners generally have positive views on the use of mHealth apps to support patients with HF management. Several challenges and opportunities for app adoption were also identified. HCPs are gatekeepers of health care delivery; thus, they are an integral part of the successful adoption and implementation of mHealth technologies in practice. Although HCPs may not be the primary users of mHealth apps, their views on these apps’ perceived advantages and their degree of compatibility with patient care and needs combined with the HCPs’ unique understanding of what is required to support patients in HF management will influence patients’ decision to use such apps for the management of their condition. Our findings support the importance of including the perspectives of HCPs, who are key stakeholders in integrating such technologies into routine clinical practice, in the development and implementation of mHealth apps.

## Appendix

Multimedia Appendix 1 Interview guide.

## Abbreviations

COREQ: Consolidated Criteria for Reporting Qualitative Research
HCP: health care provider
HF: heart failure
mHealth: mobile health

## Data Availability

The data sets generated and analyzed during the course of conducting this study are available from the corresponding author on reasonable request.
