# Supplementary material for: Evaluating Health Care Provider Perspectives on the Use of Mobile Apps to Support Patients With Heart Failure Management: Qualitative Descriptive Study
Source: JMIR Cardio. 2022 Oct 26;6(2):e40546. doi: 10.2196/40546 (PMC9647459; doi:10.2196/40546)
Supplement: Multimedia Appendix 1 [file cardio_v6i2e40546_app1.pdf]

Interview Guide

Relative advantage

Q1) What are your thoughts on using eHealth technologies, such as a mobile app to manage heart failure, specifically with helping patients adhere to their dietary and medication regimes?

Compatibility

Q2) How can using an app for heart failure management be compatible with the goals of patient care?

Complexity

Q3) What content areas do you feel that an app aiding in heart failure management should focus on for patients?

Q4) What behavioral change strategies *(ways to motivate change in behaviour)* do you feel would be effective in helping patients adhere to their dietary and medication regimes? Why or why not?

Q5) If there was an app that had an interface for patients and healthcare providers to share, what would be some of the benefits and challenges to this?

Trialability

Q6) Do you feel an app for heart failure management is something your patients would use?

Observable results

Q7) From your perspective, why would some patients use an app for heart failure management? Why would some patients choose not to use an app for managing heart failure?

Q8) From your perspective, if there was an interface for patient and healthcare providers to share in the heart failure app, why would you use this app or why would you not use this app to aid in providing care for your patients?
